# Avoidable Accidents

**Published:** 1911-05-13

**Authors:** R. C. Craven

**Affiliations:** Whitworth Park, Manchester


### Institutional Letter-Box.

### AVOIDABLE ACCIDENTS.

*To the Editor of* The Hospital.

Sir,—If all avoidable accidents were guarded against by reasonable precautions a good deal of work would be taken from the shoulders of hospital authorities. It is too much to hope that the time will come when there will be no preventible accidents, but there are certain lines on which reform can and ought to be undertaken.

Notwithstanding the fact that fireguards have come into almost general use, it is remarkable that there is no apparent diminution in the number of burning accidents. This is due very largely to the fact that the great bulk of the people clothe themselves in highly-inflammable fabrics. The price of woollen materials of a quality likely to ensure durability is high, being something like three times as much as the most general competitor of woollen goods—flannelette. Flannelette is apparently the most dangerous fabric as an article of clothing. We have it in the report of the Coroners' Committee of the Home Office that in three months "the deaths in which flannelette played a part were no fewer than 176." The same Committee had evidence that flannelette could be made safe by a proofing process which renders the stuff permanently resistant to flame. Yet nothing is done to force manufacturers either to proof this most dangerous material or even to mark it in such a way that purchasers will know at once whether it is safe or otherwise. . . . There is more commotion about a single case of lead-poisoning, about a single railway or colliery accident than about the hundreds of flannelette victims in a whole year.

One would like to know what is the attitude of hospital authorities on this matter. Some hospitals, I know, will have no flannelette of any kind, some others insist on all flannelette supplied being flame-proof. This attitude is but logical, for hospital authorities should have nothing to do with anything connected with the creation of that suffering which they exist to mitigate.

If every hospital authority—voluntary, poor law, or otherwise—took a firm stand on the flannelette question, and refused to have any material not permanently flame-proofed, it would go far to induce flannelette makers to take a humane as well as a business view, and would lead indirectly to a diminution of the number of burning accidents.—Yours truly, R. C. Craven.

Whitworth Park, Manchester, May 2.

